# Medical ozone arrests oxidative damage progression and regulates vasoactive mediator levels in elderly patients (60–70 years) with oxidative etiology diseases

**DOI:** 10.3389/fphys.2022.1029805

**Published:** 2022-11-03

**Authors:** Olga Sonia León Fernández, Gabriel Takon Oru, Renate Viebahn-Hänsler, Gilberto López Cabreja, Irainis Serrano Espinosa, Elizabeth García Fernández

**Affiliations:** ^1^ Pharmacy and Food Institute, University of Havana, Havana, Cuba; ^2^ Medical Society for the Use of Ozone in Prevention and Therapy, Iffezheim/Baden- Baden, Germany; ^3^ National Institute of Rheumatology, Ministry of Public Health, Havana, Cuba

**Keywords:** ozone, aging, oxidative diseases, elderly people, redox status

## Abstract

Medical ozone reestablishes cellular redox balance so that it may be a valid therapeutic approach in the prevention and management of age-related diseases with oxidative etiology in older people. The aim of this study is to evaluate oxidative stress and some vasoactive substances in elderly (60–70 years) rheumatoid arthritis patients with diabetes and hypertension, as well as another group with bronchial asthma patients in order to demonstrate the beneficial effects of medical ozone in the prevention and therapy of age-related diseases in these age groups. A randomized clinical study with 45 older patients (60–70 years) was performed. Group I (n = 15) with rheumatoid arthritis + diabetes and hypertension received no ozone treatment, and group II (n = 30) was treated with medical ozone. This group was divided into two subgroups (n = 15 each), group IIa: the same as group I + medical ozone and group IIb: bronchial asthma patients. Indicators of RA in I and IIa groups were evaluated. Redox balance was assessed through defense and injury biomarkers. Thromboxane A2 (TXA2) and prostacyclin levels were assessed in group IIb patients. Medical ozone arrested oxidative injury progression in the Ia group and decreased thromboxane levels and the TXA2/6-keto PGF1α ratio in the IIb group. Medical ozone arrested the progression of oxidative damage and modulated those endogenous mechanisms that promote a suitable redox status and TXA2/PGI2 balance. These results suggest that medical ozone may become a standard approach in the prevention and management of age-related oxidative diseases in elderly people.

## 1 Introduction

Aging is a growing global problem with a direct impact on society. In the 20th century, a life expectancy of 48 and 51 years was estimated for men and women, respectively, increasing to 76 and 81 years in 2017 (Xu, 2020). During aging, tissues and organs gradually start losing their functions (Flatt, 2012).

The theory of oxidative stress on aging is based on the loss of functions associated with age, which is the result of the accumulation of oxidative damage to proteins, lipids, and DNA. Although the exact mechanism of aging induced by oxidative damage is not completely clear, it is considered that the increase in the generation of reactive oxygen and nitrogen species (ERON) leads to irreversible cell senescence, which involves the release of cytokines, chemokines, growth factors, and metalloproteases among other components (Pole et al., 2016). On the other hand, elevated levels of ERON can promote life-threatening conditions such as cardiovascular diseases (Guo and Ward, 2007), rheumatoid arthritis (Mahajan and Tandon, 2004; Hajam et al., 2017), neurological diseases (Allan Butterfield, 2002), disorders of the liver, kidney, and reproductive system, and diabetes (Reichmann et al., 2018).

Medical ozone is an ozone/oxygen mixture administered at low concentrations. It can reestablish the cellular redox balance and regulate different mediator levels through an ozone oxidative pre-/post-conditioning mechanism (León et al., 1998). The proposed mechanism has been validated in pathological conditions such as ischemic syndrome, diabetes and diabetic foot, disc hernia, rheumatoid arthritis, and other diseases (León, 2014; León et al., 2016) commonly found in elderly patients.

Although aging seems to be inevitable and irreversible, recent findings on the molecular mechanisms involved in this biological process show that aging can be considered a disease and, as other diseases, it can be prevented and is potentially treatable (Undurti, 2018).

This background constituted the basis of this research work; so, its aim is to evaluate the oxidative stress and some vasoactive substance concentrations in elderly (60–70 years) rheumatoid arthritis patients with diabetes and hypertension comorbidities, as well as another elderly group of patients with bronchial asthma, in order to know the beneficial effects of medical ozone in the prevention and therapy of diseases in these age groups.

## 2 Material and methods

### 2.1 Study design

This randomized controlled clinical study was approved by the joint institutional review board (Scientific and Ethics Committees of the National Institute of Rheumatology, Ministry of Public Health, Cuba, and the Pharmacy and Food Institute, University of Havana, Cuba) in accordance with the principles of the Declaration of Helsinki (World Medical Association Declaration of Helsinki, 2011). All patients gave their informed consent for enrollment after receiving adequate information concerning the study (characteristics of the study, benefits, and possible side effects). Before enrollment, all participants attended a training program to familiarize them with the study objectives and treatment plans. The personnel involved emphasized that all participating physicians would treat each patient according to the randomized scheme of treatment through a Research Randomizer form (Randomizer, 2022). The patients were randomized into two different groups of treatment: group I (n = 15), without ozone (who received no medical ozone), and group II (n = 30), with ozone (medical ozone introduced *via* rectal insufflation).

Group IIa (n = 15), including elderly patients (60–70 years) with rheumatoid arthritis (RA) + diabetes and hypertension as comorbidities, and group IIb, including elderly patients (60–70 years) with bronchial asthma (BA) (n = 15), participated in progress research in the context of the effects of medical ozone on vasoactive substances. These patients fulfilled the same ethical protocol as group IIa. In this protocol, each patient was his/her own control (i.e., before medical ozone treatment).

Inclusion criteria: I and IIa groups. Elderly patients (60–70 years) of both sexes and different ethnic origins with a diagnosis of RA who fulfilled the revised American Rheumatism Association’s (Arnett et al., 1988) criteria for RA (morning stiffness, swelling of hand joints, swelling of three or more joints, and symmetric swelling of joints) were eligible to participate in the study. Patients of the National Institute of Rheumatology, Ministry of Public Health, Cuba, who fulfilled the following criteria were chosen: disease activity score 28 (DAS 28 > 3.2 and 5.1), whose examination was carried out under blinded conditions by a physician different to the one who selected the patients according to a randomized scheme of treatment and a brief medical history; the Health Assessment Questionnaire–Disability Index (HAQ-DI, according to the validated Spanish version) (Cardiel et al., 1993): patients with a disease duration longer than 5 years were included. Exclusion criteria: patients with any history of chronic conditions such as liver disease, respiratory disorders, and alcohol usage and smoking were not included in the study; and patients with overlapping syndromes, cancer, or other associated autoimmune disorders or who were pregnant were also excluded. Inclusion criteria: group IIb patients were required to meet the following conditions: 1) elderly patients (60–70 years), 2) diagnosed with asthma for at least 6 months, and 3) treated with an inhaled corticosteroid and oral or inhaled beta-2 agonist. For 15 selected patients, specifically, clinical data were collected, including spirometry levels (measures the rate of airflow and estimates the lung size), chest X-ray, and allergy-related conditions. Exclusion criteria: patients who were 1) diagnosed with respiratory infections or immune deficiency requiring specific therapy or any other diseases that may influence the asthma evolution, 2) smokers, 3) under alcohol usage, and 4) in systemic steroid therapy throughout the preceding 2 weeks were excluded.

Medical ozone was generated using an OZOMED unit, Cuba. A total of 20 treatments by rectal insufflations (five/week from Monday to Friday) were performed. Next, 20–30 mg/l of ozone was administered in a stepped application and in an increasing order as follows:

First week: 20 mg/l, 100 ml; second week: 25 mg/l, 100 ml; third week: 25 mg/l, 150 ml; and fourth week: 30 mg/l, 200 ml.

Medical personnel were instructed to report all adverse reactions, whether described in the package circulars of the study medications or not.

### 2.2 Biochemical determinations

Blood samples for biochemical analysis were obtained after a 12-h overnight fast in the beginning and 24 h after the final ozone treatments for I, IIa, and IIb groups.

Redox parameters were determined by spectrophotometric methods using a BOECO Model S-220 Spectrophotometer, Germany. The superoxide dismutase (SOD) activity was measured using kits supplied by Randox Laboratories Ltd., Ireland (Cat. nos. SD125 and RS505). The catalase (CAT) activity was measured by following the decomposition of hydrogen peroxide at 240 nm at 10 s intervals over 1 min (Biochemica Information Boehringer Mannheim, 1987). After precipitation of thiol proteins using 10% trichloroacetic acid, reduced glutathione (GSH) was measured according to the method of Sedlak and Lindsay (1968a) with Ellman’s reagent [5′5 dithiobis-(2-nitrobenzoic acid) 10-2 M (Sigma St. Louis, MO, United States)]; absorbance was measured at 412 nm. Nitrite/nitrate levels as a measure of nitric oxide (NO) were determined by the Griess reaction after first converting nitrates to nitrites using nitrate reductase (Boehringer Mannheim Italy SpA, Milan, Italy) (Granger et al., 2022). The advanced oxidation protein products (AOPP) were measured as the oxidation of the iodide anion to diatomic iodine by advanced oxidation protein products (Wirko-Sarsat et al., 2022). Quantification of total hydroperoxides (TH) was measured by using a Bioxytech H_2_O_2_-560 kit (Oxis International Inc., Portland, OR, United States). Concentrations of malondialdehyde (MDA) were analyzed using the LPO-586 kit obtained from Calbiochem (La Jolla, CA).

Analysis of prostanoids: Prostanoid plasma specimens (TXB_2_ and 6-keto PGF_1α_) were extracted using C18 reversed-phase cartridges (Sep-Pak cartridges) (Powell et al., 1982).

After evaporation of the organic solvent eluates, dry residues were resuspended for subsequent HPLC-RIA determinations (Gelpí et al., 1989) in acetonitrile/0.04 M formic acid with triethylamine buffer at pH 3.15 (33/76 v/v).

Previously, recoveries for TXB_2_ and 6-keto PGF_1α_ were established by loading Sep-Pak cartridges with 1 ml of plasma supplemented with a known amount (0.02 µCi) of 3H-labeled standards. HPLC recoveries were estimated directly from the counts in each of the fractions collected at the retention times of the prostanoids. Recoveries (%) after Sep-Pak extraction and HPLC purification were 69 ± 5 for TXB_2_ and 70 ± 4 for 6-keto PGF1α (mean ± SD).

### 2.3 Statistical analysis

The OUTLIERS preliminary test for detection of error values was initially applied. Afterward, data were analyzed by one way analysis of variance (ANOVA), followed by a homogeneity variance test (Bartlett–Box). In addition, a multiple comparison test was used (Student–Newman–Keuls test). Student’s t-test for independent samples, canonical discriminate analysis, and *t*-test of paired samples were applied. The results are presented as means ± standard error of the mean (S.E.M). The level of statistical significance used was at least *p* < 0.05.

## 3 Results

### 3.1 Characteristics of the patients involved in the study and the clinical view of RA patients + comorbidities

In relation to the baseline characteristics (Table 1), the groups were similar at randomization (*p* > 0.05). Women were the predominant sex in groups I and IIa, while similar sexes in group IIb were found. Age (60–70 years) displayed no differences among groups. An increase in Caucasian patients in I and IIa groups was observed. In group IIb, the number of Caucasians was similar to that of non-Caucasians.

**TABLE 1:** Clinical images of older patients (60-70 years) with oxidative etiology diseases.

Demographic data/patient history	Group I without ozone (n = 15)	Group II with ozone (n= 30)	
		Group IIa (n =15)	Group IIb (n =15)
Group IIa: rheumatoid arthritis + comorbidities		15/100%	-
Women (n/%)	12/80%	10/67%	-
Men (n/%)	3/20%	5/33%	-
Age (years)	63 ± 1^(a)^	62 ± 1^(a)^	-
Comorbidities			-
Diabetes mellitus	13/86%	14/93%	-
Hypertension	14/93%	15/100%	-
Group IIb: bronchial asthma	-	-	15/100%
Women (n/%)	-	-	8/53%
Men (n/%)	-	-	7/47%
Age (years)	-	-	65 ± 3^(a)^
Previous therapy			
Methotrexate (MTX)			
Ibuprofen			
Folic acid			
Other drugs (oral and inhalation routes)			
Hypoglycemic (metformin)			
Anti-hypertensive (enalapril + hydrochlorothiazide)			
Bronchodilators (corticosteroid and B2 agonists)			
Race			
Caucasian	13/87%	10/66%	9/60%
Non-Caucasian	2/13%	5/34%	6/40%

**Group I:** MTX + ibuprofen + folic acid + hypoglycemic/anti-hypertensive drugs.

**Group IIa**: **rheumatoid arthritis + comorbidities**: the same basic treatment as group I + medical ozone).

**Group IIb**: **bronchial asthma:** bronchodilators + medical ozone (each patient was his/her own control (i.e., before medical ozone treatment)).

The data reflecting the age are the mean ± S.E.M. in each group. Mean values with different letters indicate significant differences (*p* < 0.05) between both groups.

**FIGURE 1:**
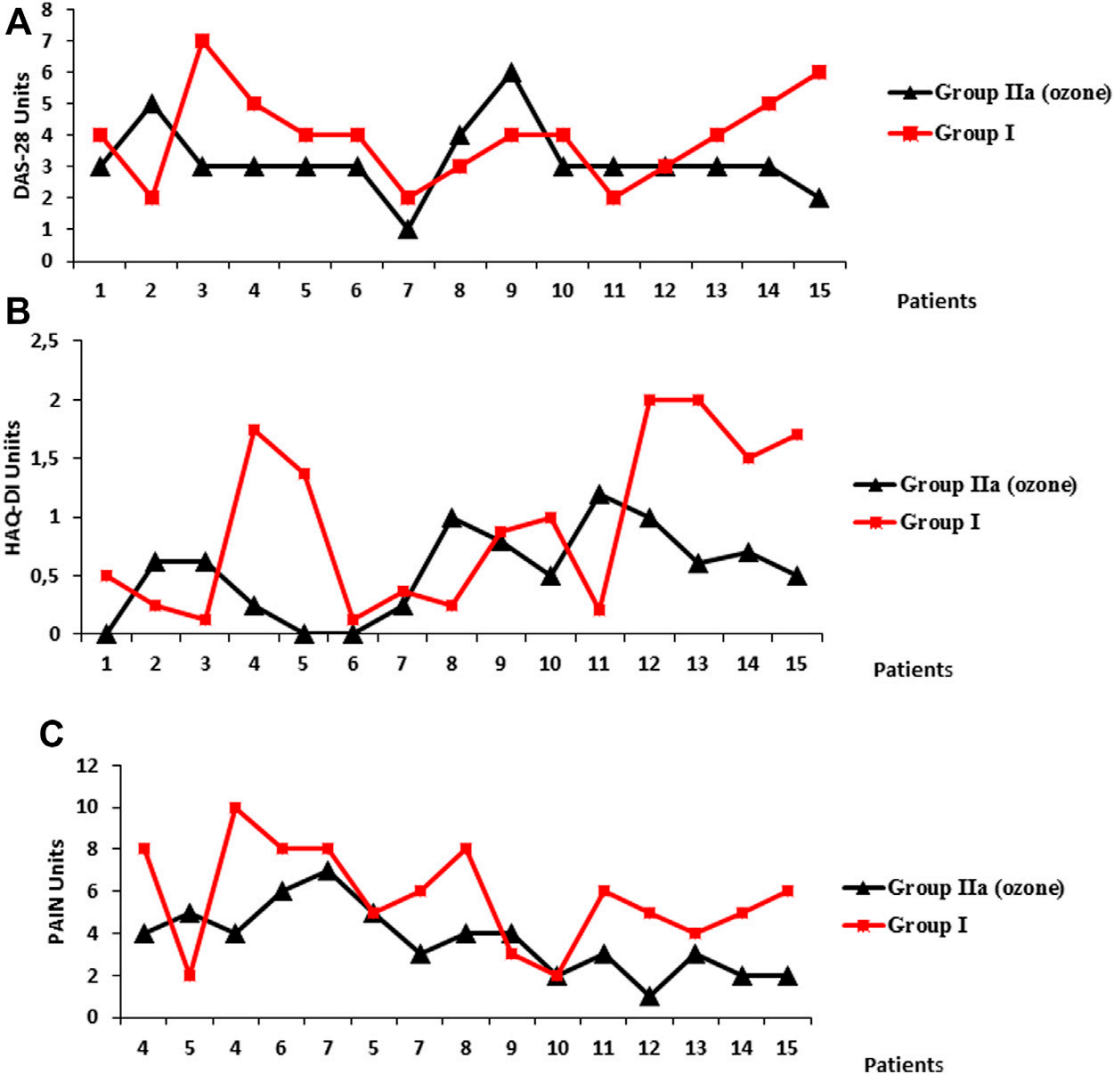
Clinical variables: **(A)** DAS28 (Disease Activity Score 28), **(B)** HAQ-DI (Health Assessment Questionnaire–Disability Index), and pain **(C)** (Visual Analogical Scale, VAS) in two groups of elderly patients (60–70 years (n = 30). Group I, without medical ozone (n = 15) and group IIa, rheumatoid arthritis + comorbidities, n = 15, treated with medical ozone + methotrexate + ibuprofen + folic acid. Group I (same as group IIa but without medical ozone. DAS28, low activity ≤3.2; moderate activity >3.2; and ≤5.1: high activity > 5.1 HAQ-DI, (+ >1.25). Pain, Visual Analogical Scale (VAS) from “0” (minimum pain intensity) to “10” (maximum pain intensity). No pain was considered “0.” Statistical analysis for the variables showed the following results: DAS28, group IIa (end of the experiment) vs. group I. **3** ± 0.2 vs. **5** ± 0.4 (p < 0.05) HAQ-DI, group IIa (end of the experiment) vs. group I. **0.64** ± 0.1 vs. **1.3** ± 0.2 (p < 0.05) pain, group IIa (end of the experiment) vs. group I. **3** ± 0.4 vs **5** ± 0.1 (p < 0.05).

After medical ozone treatment, an improvement in DAS28, HAQ-DI, and the Pain Index was observed (Figure 1). (A) 80% of patients showed low-activity disease (group IIa) *versus* 4% in patients who received no ozone treatment (group I). (B) There was a correspondence with the HAQ-DI (disability index) as in group IIa, 94% were negative compared with 60% in group I (+>1.25 points), and with regard to pain (C), 80% of patients who were treated with medical ozone had more than 50% pain perception reduced in the VAS *versus* 47% of patients without ozone therapy.

### 3.2 Redox biomarker levels in groups I (without ozone) and IIa (before and after ozone treatment)

**FIGURE 2:**
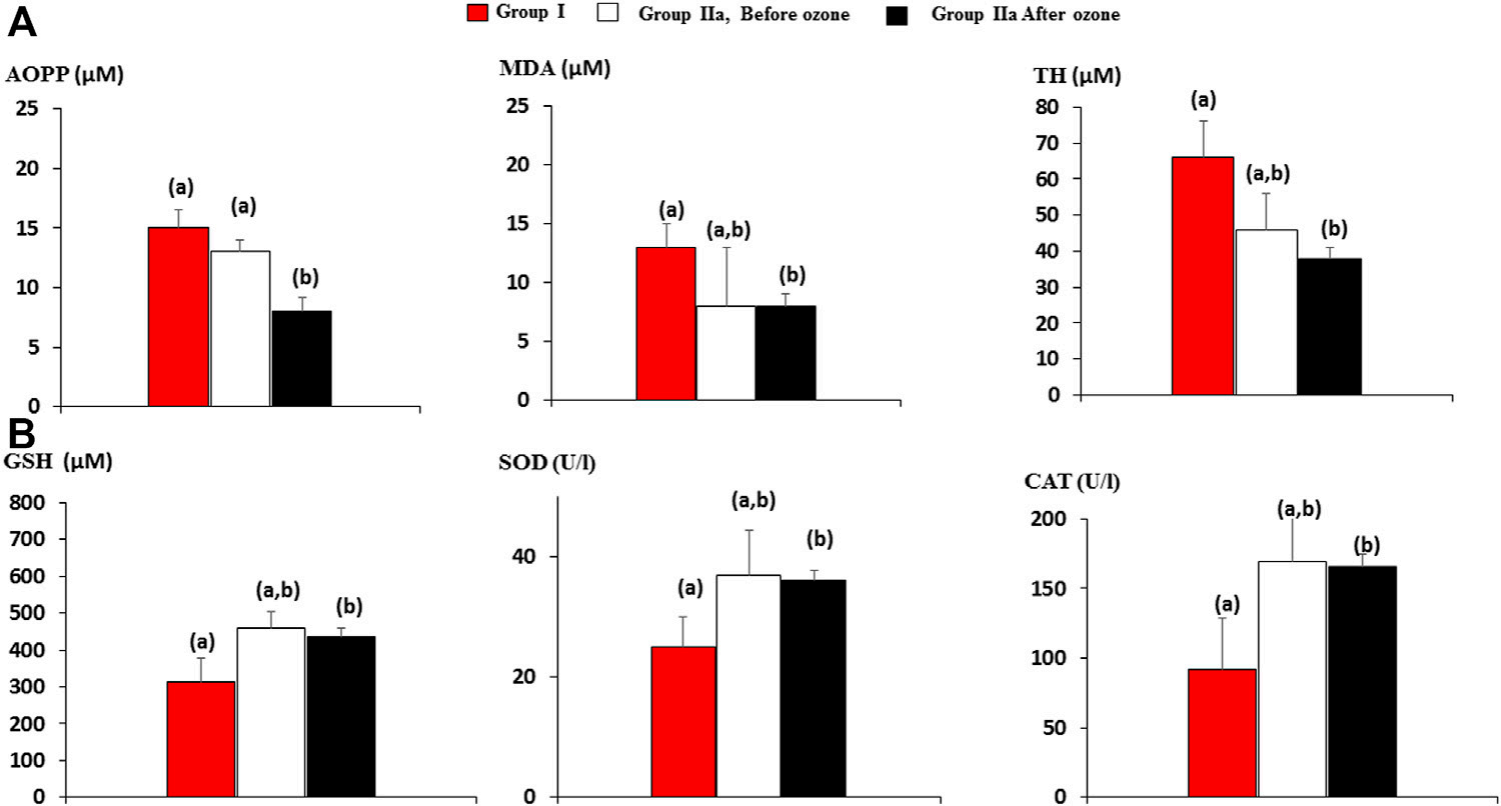
Injury and antioxidant defense biomarkers in older patients (60–70 years old) with rheumatoid arthritis + comorbidities in group I (n = 15) and group IIa, rheumatoid arthritis + comorbidities, n = 15, treated with medical ozone + methotrexate + ibuprofen + folic acid. Group I, the same as IIa, but without medical ozone. **(A)** Injury markers, AOPP, advanced oxidation protein product; MDA, malondialdehyde; TH, total hydroperoxide; **(B)** antioxidant defense markers, GSH, reduced glutathione; SOD, superoxide dismutase activity; CAT, catalase activity. The data reflecting AOPP, MDA, TH, GSH levels, SOD, and CAT activities are the mean ± S.E.M. of each group. Mean values with different letters indicate significant differences (*p* <0.05) between groups.

Plasmatic determinations of antioxidant defenses and injury redox markers in both groups of patients were studied (Figure 2).

After ozone treatment, no change in injury (A) and antioxidant defense (B) biomarkers could be observed except protein damage (AOPP), which decreased significantly after medical ozone treatment. Nevertheless, when the redox biomarkers after ozone treatment were compared with those of group I (without ozone therapy), then statistical differences were found. Antioxidant defenses (GSH, SOD, and CAT) increased, whereas injury biomarkers (MDA and TH) decreased compared with group I.

It is important to emphasize that there were no differences between I (RA + diabetes and hypertension) and IIa (medical ozone + RA + diabetes and hypertension) groups before ozone; therefore, these results suggest that, in elderly patients (60–70 years) with RA + comorbidities, medical ozone arrests the progression of oxidative damage accompanied by disease improvement.

### 3.3 Nitric oxide and other vasoactive mediators in elderly patients with RA + comorbidities and bronchial asthma

Nitric oxide is a very important and controversial molecule with vasodilator effects which play an important role in different diseases with oxidative etiology and regulation of signaling mechanisms.

**FIGURE 3:**
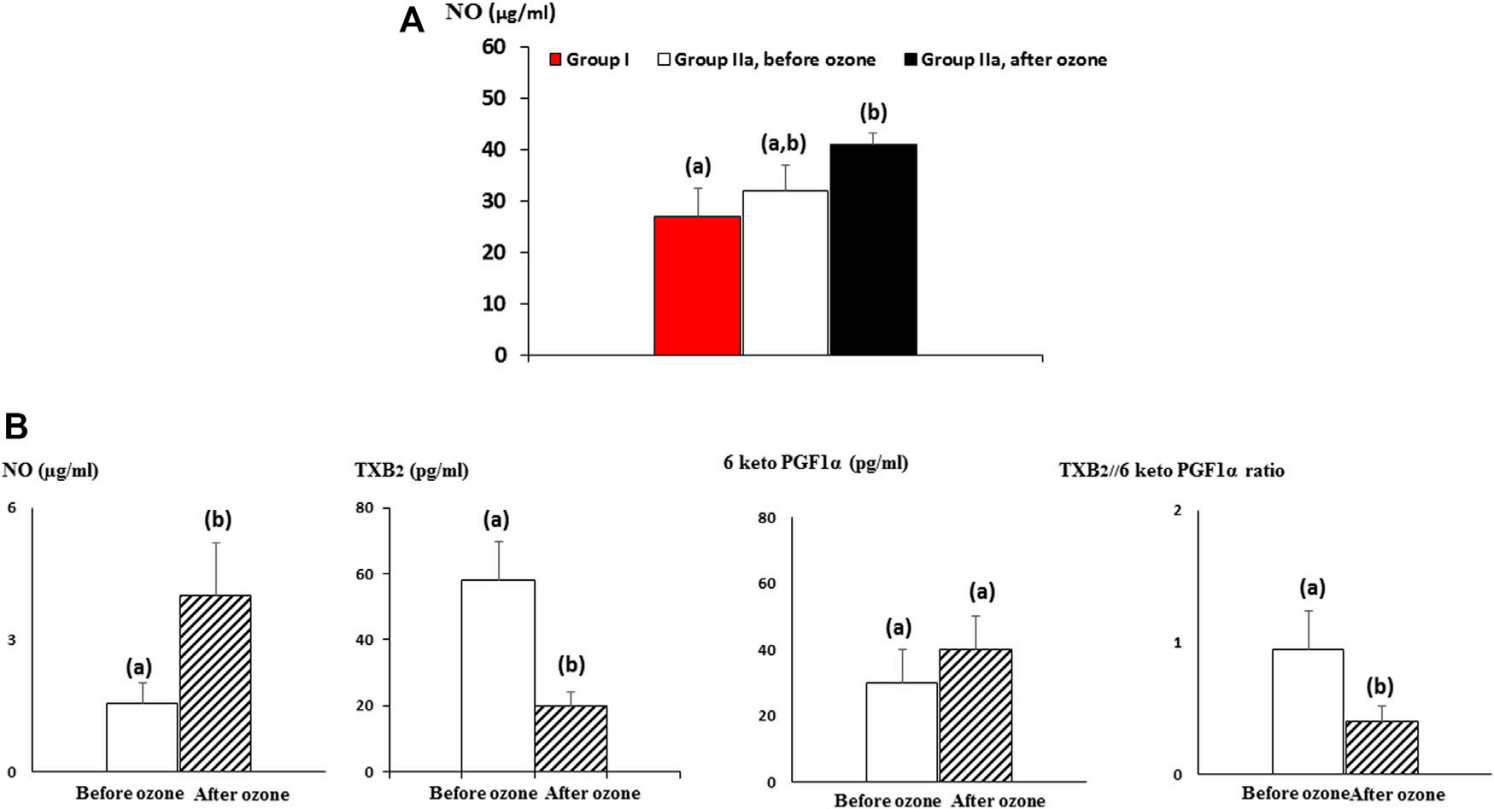
Medical ozone effects on vasoactive mediators in older patients (60–70 years old). **(A)** NO (nitric oxide) levels in rheumatoid arthritis + comorbidities patients. **(B)** NO, TXB2 (the stable metabolite of TXA2), and 6-keto PGF1α (the stable metabolite of prostacyclin) plasma concentrations in bronchial asthma patients before and after ozone treatment. The data reflecting NO, TXB2, and 6-keto PGF1α plasmatic levels are the mean ± S.E.M. of each group. Mean values with different letters indicate significant differences (*p* <0.05) between groups.

NO in group IIa (RA + comorbidities) showed a similar view to antioxidant defenses and injury biomarkers. No differences before and after ozone treatment were found although medical ozone increased (*p* < 0.05) NO concentrations with regard to group I (patients without ozone treatment) (Figure 3A).

Bronchial asthma is characterized by bronchoconstriction, for which the vasodilatory effects of NO represent an improvement in the clinical conditions of these older patients (Figure 3B).

Prostanoids are bioactive lipid mediators and consist of prostaglandins, prostacyclin, and thromboxane. These biomolecules are widely distributed throughout the body, regulating important physiological and physiopathological processes. Thromboxane A2 (TXA_2_), with prothrombotic properties (Krüger-Genge et al., 2018), is mainly produced in platelets and endothelial cells, while prostacyclin (PGI2) is synthesized fundamentally in endothelial cells and smooth muscle cells. Both prostanoids are unstable with half-life times of 5–7 min for TXA2 and plasmatic concentrations that range between 2 and 285 pg/ml (Simmons et al., 2004). Prostacyclin has an approximate half-life of 42 s after which it is transformed (not enzymatically) to 6-keto PGF_1α_ (Chu et al., 2015; Lewis and Dollery, 1983). The vasodilator effects of prostacyclin counteract the prothrombotic and vasoconstriction actions of thromboxane. Prostacyclin inhibits platelet activation (Borgdorff et al., 2006) with a major inhibitory potential than that of other prostanoids such as PGD_2_ and PGE_2_ (Whittle et al., 1978).

Figure 3B shows medical ozone effects on TXB2, 6-keto PGF1α plasma levels, and the relationship between them in older patients (60–70 years) with bronchial asthma before and after ozone treatment. A decrease in TXB2 (*p* < 0.05) and an increased tendency in the formation of the stable metabolite of prostacyclin (6-keto PGF1α) were observed, whereas the ratio displayed a significant reduction (*p* < 0.05) after ozone treatment. This suggests that medical ozone promotes vasodilatation predominance *versus* vasoconstriction in these patients.

## 4 Discussion

Medical ozone is a pleiotropic therapeutic concept. It regulates different mediators with a common characteristic: their close association with the cellular redox status. Medical ozone was able to arrest injury oxidative progression in older patients with RA + comorbidities. These results are closely related to a reduction in the total hydroperoxide levels, including hydrogen peroxide, a reduction of protein and lipid damage, and an increase in SOD and CAT activities.

The increase in ERON generation leads to a decrease in their scavenger systems, a pro-oxidant redox state, and cell aging (López-Lluch et al., 2015). The decrease in superoxide and catalase activities of mitochondrial origin increases oxidative stress in mice and leads to premature aging and pathological conditions characteristic of aging (Panday et al., 2015). The reduction of TH and protein damage suggests the decrease of cartilage destruction in patients with RA + comorbidities treated with medical ozone which is in line with DAS_28_ results and HAQ-DI.

Hydrogen peroxide is capable of crossing cell membranes from its generation site toward the target molecule (Lewis and Dollery, 1983), fundamentally damaging –SH groups from cysteine protein residues (Corcoran et al., 2013). Oxidative stress is associated with the biochemical signaling mechanisms involved in aging. One of these mechanisms is mitochondrial dysfunction that leads to an increase in the production of ROS, fundamentally superoxide radicals, and hydrogen peroxide (Sies, 2017). A deficit in SOD increases superoxide radical levels, whereas an excessive SOD activity leads to hydrogen peroxide overproduction which may be reduced if CAT is activated. In fact, medical ozone regulated these important redox biomarkers.

H_2_O_2_ is considered a second messenger along with other ROS (Veal et al., 2007). It is able to activate cellular metabolic responses which can influence different essential cell events such as proliferation, survival, or death (Younis et al., 2022). Likewise, the excessive production of hydroxyl radicals through the Haber–Weiss reaction as a result of the increase of hydrogen peroxide and superoxide radicals is able to promote the peroxidation of lipids with damage to the cellular membrane and lipoproteins. As a result, malondialdehyde (MDA) and conjugated dienes are formed, both of these known as cytotoxic and mutagenic compounds. The rate of lipid peroxidation increases very rapidly and affects a huge quantity of lipid molecules (Nishida et al., 2013).

Mitochondrial dysfunction plays an important role in the aging process of cells which involves a reduction of the life span of cells due to a functional inefficiency of enzymes, proteins, and other biomolecules, also accelerating the peroxidative damage to membrane lipids (Bertram and Hass, 2007). Mitochondrial DNA is a target molecule in this process. Unlike cellular DNA, located in the core of the cell and surrounded by a membrane that protects it against damage by ERON, mitochondrial DNA lacks nucleosomes and repair mechanisms which makes it highly sensitive to oxidative damage during aging (Mikhed et al., 2022). This process, together with the reduction of antioxidant defense mechanisms, with the advance of age, contributes to accentuate the adverse effects of oxidative stress with aging (Lobo et al., 2013). The genotoxicity induced by an overproduction of ROS leads to DNA fragmentation. However, this effect can be abolished or attenuated by the action of proteins belonging to the family of Hsp70. It has been reported that the heat shock proteins work hand-in-hand with the antioxidant system to inhibit or neutralize the cellular effects of ROS (Wu et al., 2015). In line with these results, immunohistochemical studies showed that medical ozone regulates Hsp70 expression in liver ischemia/reperfusion injury (León Fernández et al., 2008) by strengthening the antioxidant status when medical ozone is used as a form of treatment.

Diabetes and hypertension are comorbidities associated with the older patients included in this study. Both are vascular dysfunctions which have, as a molecular base, an imbalance between ROS production and antioxidant defense, progressive with aging.

The aging of the vascular system is associated with the morbidity and mortality of older people. In order to develop novel treatments for the amelioration of vascular aging and the prevention of age-related vascular pathologies, it is essential to understand the cellular and functional changes that occur in the vasculature during aging (Ungvari et al., 2018).

Four pathological pathways have been proposed in order to explain diabetes vascular complications: polyol, hexosamine, PKC (protein kinase C), and advanced glycation end product (AGE) pathways. All of them have a critical point of inhibition at the glyceraldehyde 3-phosphate dehydrogenase (GAPDH) level by a free radical: superoxide radical anion (Brownlee, 2001). When the enzyme is inhibited, all intermediary metabolites of the glycolytic pathway are accumulated, and they activate the four aforementioned pathways, each of which leads to vascular dysfunctions.

In an experimental diabetes model in rats and a clinical trial of patients with diabetic foot, medical ozone increased the superoxide dismutase activity with a reduction in oxidative stress and markers of endothelial damage (Al-Dalain et al., 2001; Martínez-Sánchez et al., 2005). In this study, RA + comorbidity in older patients increased the superoxide dismutase activity compared to patients receiving no ozone, constituting a contribution to the arrest of oxidative injury progression. These results suggest that superoxide radicals are captured by the SOD enzyme, consequently decreasing pathological route activation and vascular dysfunctions.

Hypertension is closely connected with an overproduction of ROS and endothelial damage, so an improvement in the redox status (especially NO and superoxide radicals) should ameliorate the imbalance of blood pressure.

Hypertension is associated with oxidative stress and serum concentrations of several vasoconstriction agents such as angiotensin II and others. The increase in ROS production, fundamentally the radical superoxide anion, is mediated by the increase of NADPH oxidase (NOx) (Nguyen Dinh Cat et al., 2013).

Therefore, endothelial and vascular smooth muscle NADPH oxidase-dependent ROS are strongly implicated in such hypertension-related vascular dysfunction. In addition, the increase in ROS production exerts a profound influence on different systems that regulate blood pressure (Higashi et al., 2012). Evidence from experimental studies indicates that hypertension is associated in a complex manner with ROS generation and that a cause-and-effect relationship exists between the two (Montezano and Touyz, 2014).

The vascular tone is regulated by physiological oxidative stress through the activity of the SOD-dependent copper/zinc and the levels of superoxide and hydrogen peroxide. It has been reported that the increase in vascular resistance and the constriction of the blood vessels lead to hypertension.

Vasoactive peptides, including angiotensin II and thromboxane A2, play critical roles in the pathological mechanistic insight into the occurrence and progression of hypertensive disorders in humans (Touyz et al., 2018). The results indicate Nox1 involvement in increased thromboxane A2 synthase expression in human vascular smooth muscle cells, underlining the close relationship between oxidative stress and prostaglandin release.

Bronchial asthma is a complex process that involves the interrelation of different factors of the host, such as innate immunity, genetic aspects, and sex itself, as well as exposure to environmental stimuli such as allergens and infections. The activation of phagocytic cells and inflammatory mediators in the airways of asthmatic patients leads to high levels of oxidative stress in the lungs (Al- et al., 2011; Montezano and Touyz, 2014). This increased oxidative stress affects mucus hypersecretion and alters the capillary endothelium, which may cause a leak of reactive oxygen species (ROS) into the systemic circulation (Nadeem et al., 2014).

In asthma patients treated with medical ozone, improved bronchoconstriction and peak expiratory flow were associated with a decrease in TXB_2_, as well as in the TXB_2_/6-keto PGF1α ratio. It has been reported that the levels of some prostanoids (PGD2, TXB2, and PGI2) were much higher (up to 208-fold) in the bronchoalveolar fluid 5 min after an allergen challenge in allergic asthmatic patients shared with controls (Liu et al., 1991). As already mentioned, TXA2 is a powerful bronchoconstrictor, and in asthmatic patients, high concentrations of this prostanoid have been observed. This suggests the possible role of TXA2 in airway remodeling or hyper-responsiveness (Davi et al., 2014). Such an effect was mediated through c-Jun N-terminal kinase–mitogen-activated protein kinase signaling, and this increased the extracellular calcium influx after stimulation of the TXA2 receptor (Lei et al., 2014).

By contrast, prostacyclin counteracts the negative effects of TXB2. It has been shown that in murine experimental models, PGI2 analogs (iloprost) reduced the maturation and migration of dendritic (DC) cells from the lungs. These cells participate in the specific Th2 response of the allergen in lymphatic nodes (Idzko et al., 1991), which is why a modulation of the immune response occurs. Additionally, T-cell differentiation was reduced in effector Th2 cells (Zhou et al., 2007). Another important effect of prostacyclin is its capacity, as well as PGE2, to act as an immune suppressor in eosinophils (Peinhaupt et al., 2017). The actions of PGI2 on eosinophils are diverse: it inhibits trafficking of eosinophils in the bone marrow (Sturm et al., 2011) and modulates eosinophil–endothelial interactions by the inhibition of eosinophil adhesion and transendothelial migration (Konya et al., 2010). It is important to emphasize the effects of medical ozone on TXA2 and PGI2 in diabetes.

Hyperglycemia activates the expression of COX-2, an enzyme that catalyzes the formation of intermediary endoperoxides and the formation of TXA2 with a reduction in the formation of prostacyclin and nitric oxide, leading to oxidative stress induced by hyperglycemia.

Thus, there is a close interaction between the COX-2-PG system, hyperglycemia-induced oxidative stress, and NO release (Consentino et al., 2003). These events are counteracted by medical ozone as this study has demonstrated.

In order to maintain ozone´s beneficial effects after the first cycle (20 ozone treatments), it is suggested the patients should receive a second cycle with an interval of 3 months. Potentiation of clinical and redox responses after a second ozone exposure has been demonstrated (Takon Oru et al., 2022; Konya et al., 2010; Sturm et al., 2011; Takon Oru et al., 2022), so ozone protective effects could be prolonged or even improved.

In summary, medical ozone arrested oxidative damage progression in elderly patients through the regulation of SOD, hydrogen peroxide, and catalase which lead to a decrease in protein and lipid damage. On the other hand, medical ozone was able to modulate those endogenous mechanisms that promote a suitable redox status and TXA2/PGI2 balance. In addition, ozone not only arrests oxidative injury progression but it also acts on the comorbidities of RA *via* the regulatory action of ozone on glucose metabolism and vascular damage. However, these findings need to be verified with prospective investigations which are in progress. These results suggest that medical ozone may become a standard approach in the prevention and management of age-related oxidative diseases which have, up until now, not only been frequent but also have constituted many risks for elderly people.

## Data Availability

The raw data supporting the conclusions of this article will be made available by the authors, without undue reservation.
